# Atlas of Prenatal Hair Follicle Morphogenesis Using the Pig as a Model System

**DOI:** 10.3389/fcell.2021.721979

**Published:** 2021-10-07

**Authors:** Yao Jiang, Quan Zou, Bo Liu, Shujuan Li, Yi Wang, Tianlong Liu, Xiangdong Ding

**Affiliations:** ^1^National Engineering Laboratory for Animal Breeding, Laboratory of Animal Genetics, Breeding and Reproduction, Ministry of Agriculture, College of Animal Science and Technology, China Agricultural University, Beijing, China; ^2^Institute of Animal Husbandry and Veterinary Medicine, Anhui Academy of Agricultural Sciences, Hefei, China; ^3^Key Laboratory of Animal Epidemiology of the Ministry of Agriculture, College of Veterinary Medicine, China Agricultural University, Beijing, China

**Keywords:** pig, hair follicle, hair types, morphogenesis development, animal model

## Abstract

The pig is an increasingly popular biomedical model, but only a few in depth data exist on its studies in hair follicle (HF) morphogenesis and development. Hence, the objective of this study was to identify the suitability of the pig as an animal model for human hair research. We performed a classification of pig HF morphogenesis stages and hair types. All four different hair types sampled from 17 different body parts in pig were similar to those of human. The Guard_2 sub-type was more similar to type II human scalp hair while Guard_1, Awl, Auchene, and Zigzag were similar to type I scalp hair. Based on morphological observation and marker gene expression of HF at 11 different embryonic days and six postnatal days, we classified pig HF morphogenesis development from E41 to P45 into three main periods – induction (E37–E41), organogenesis (E41–E85), and cytodifferentiation (>E85). Furthermore, we demonstrated that human and pig share high similarities in HF morphogenesis occurrence time (early/mid gestational) and marker gene expression patterns. Our findings will facilitate the study of human follicle morphogenesis and research on complex hair diseases and offer researchers a suitable model for human hair research.

## Introduction

Hair follicles (HFs) are one of the most important appendages of the skin. Their development is divided into prenatal HF morphogenesis development and postnatal HF cycle development (Schneider et al., 2009). HF morphogenesis development in the prenatal stage is critically important, which determines HF cycle development in the postnatal stage. HF morphogenesis has been summarized into three distinct periods – induction (stage 0–1), organogenesis (stage 2–5) and cytodifferentiation (stage 6–8) based on their key characteristics (Paus et al., 1999). Several classical genetic markers such as *SOX2* (Saxena et al., 2019), *LEF1* (Boras-Granic et al., 2006), *SOX9* (Nguyen et al., 2018), and *KRT15* (Miyachi et al., 2018) have been established as critical genes involved in each step of morphogenesis, and studies on these genes have enabled a fine-toothed dissection of the cellular and molecular dynamics of HF morphogenesis (Sennett et al., 2015; Tomann et al., 2016).

To date, morphological and development studies that also address the genetic and molecular mechanisms underlying human HF development and structure have been conducted using mouse models providing a deeper understanding of the numerous abnormal phenotypes of the HF (Duverger and Morasso, 2014; Fuchs, 2016). These studies have, however, identified fundamental differences in the morphological structure (van Ravenzwaay and Leibold, 2004), developmental mechanisms (Porter, 2003; Duverger and Morasso, 2009; Pratt et al., 2017), and hair diseases (Porter, 2003) between human and mouse HFs. The shortage of human embryos and the ethical issues surrounding their use for research purposes have also restricted further study on human hair follicle morphogenesis during embryogenesis. Considering the above-mentioned draw-backs, a new animal model with easy availability and a high similarity to the human HF development is required to facilitate hair follicle morphogenesis research.

One such potential model, the pig, has emerged in recent years as a model for the study of numerous diseases such as atherosclerosis (Jensen et al., 2010), diabetes (Renner et al., 2010), cystic fibrosis (Rogers et al., 2008), retinitis pigmentosa (Ross et al., 2012), and cardiovascular disease (Tsang et al., 2016). A high similarity has been noted between human and pig with regard to anatomy, genetics, and physiology, and the pig is being increasingly used as an alternative research model to rodents, canines, or non-human primates (Meurens et al., 2012; Pabst, 2020; Zuidema and Sutovsky, 2020). However, very limited data are available regarding the morphogenesis and development of pig hair follicles, despite pig skin showing several anatomical and physiological similarities with human skin (Liu et al., 2010; Turner et al., 2015). Therefore, further studies are needed with regard to the suitability of using the pig as an animal model for human HF.

Thus, the objectives of this study were to evaluate the possibility of using the pig as an animal model for human HF research. In this study we have: (1) established accurate classification and recognition of HF morphogenesis stages in pig embryonic development; (2) compared morphological structures and the expression of marker genes of the specialized hair follicle of pig with human and mouse HF; and (3) classified the different hair types present in the pig skin.

## Materials and Methods

### Ethics Approval

All animal procedures were evaluated and authorized by Institutional Animal Care and Use Committee (IACUC). The whole procedure for samples collected was carried out in strict accordance with the protocol approved by the IACUC at the China Agricultural University. The IACUC of the China Agricultural University specifically approved this study (permit number DK996).

### Pig Skin Samples From Embryonic and Postnatal Stages

Yorkshire pig skin samples (1 × 3 cm) of 11 different embryonic (E) (E37, E39, E41, E43, E45, E52, E60, E85, E103, E107, E113) and six postnatal (P) days (P5, P11, P19, P22, P26, P45) were obtained from a well-defined dorsal skin region precisely located as described in the operation manual of Paus et al. (1999).

### Phenotype Measurement of Hair Shafts

**FIGURE 1:**
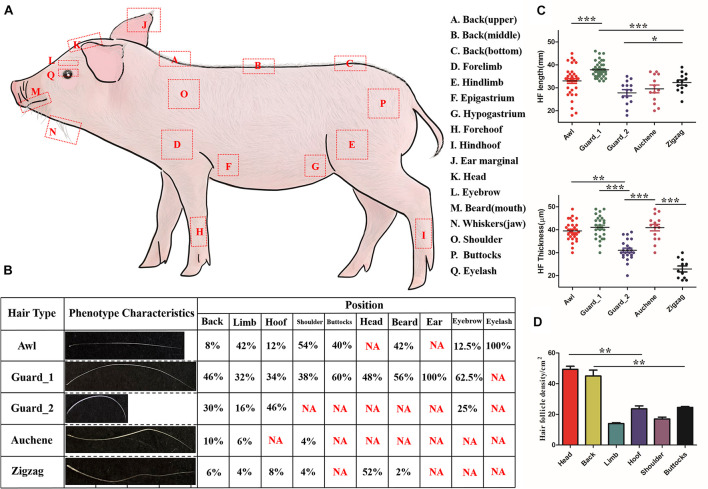
Characteristics of different hair types in pig. **(A)** Diagram illustrating the locations on the body where different hair type samples were obtained. **(B)** Five different hair types found on different body parts of pig; NA, not this hair type. **(C)** Quantification of HF length (upper panel) and HF thickness (lower panel). **(D)** Quantification of HF in different pig body parts. Each body part with three biological replications. *p < 0.05; **p < 0.01; ***p < 0.001.

Three piglets (P30) were used to perform phenotype measurement analysis. Hair shafts were sampled from 17 different body parts (Figure 1A): back (upper, middle and bottom), forelimb, hindlimb, epigastrium, hypogastrium, forehoof, hind hoof, ear marginal, head, eyebrow, eyelash, whiskers (mouth and jaw), shoulder, and buttocks. Except for the eyebrow, eyelash, and whiskers (mouth and jaw), where all the hair shafts were collected, hair shafts from the other 13 body parts within 1 cm^2^ region were collected, and three replicates were made for each body part. For each part, hair shafts were measured for hair length (mm) by a straight ruler and for hair thickness (μm) and hair density (/cm^2^) by light microscopy.

Three randomly selected images were used to perform analysis.

### Classification of Hair Types

In accordance with the classification of mouse hair types (Duverger and Morasso, 2009), pig hair shafts were classified into different hair types and summarized with length, number of waves, and bends as criteria.

### Identification of Hair Follicle Morphogenesis Development Stages

As described by Paus et al. (1999), the HF morphogenesis development stages were identified based on the characteristic derivatives of different time periods – hair placode for induction (stage 1); hair germ, DC for stage 2; hair peg, DP for stages 3–5 in organogenesis; IRS, ORS for stages 6–7; and hair shaft for stage 8 in cytodifferentiation. Hematoxylin and eosin (H&E) staining was performed to visualize the characteristic derivatives of each stage. Immunofluorescence of marker gene expression at different stages was also performed for the distinction of the different HF morphogenesis stages. Five antibodies used are listed in Table 1, and negative control (fetal bovine serum substitute antibody) result, in Supplementary Figure 1.

**TABLE 1:** The information of five antibodies in this study.

Periods	Undifferentiated	Induction	Organogenesis	Cytodifferentiation		
Stage	Stage 0	Stage 1	Stages 3–5	Stages 6–7	Stage 8	
Antibody	*LEF1*	*LEF1/SOX2*		*SOX9*	*K15*	*Ki67*
Expression region	Basal keratinocytes hair placode	Hair placode	DC/DP	IRS, ORS, hair shaft	Hair shaft, ORS	Proliferation cells
Parameter	Abcam, ab137872		Bioss, bs-23177R	Abcam, ab185966	Santa, sc-47697	CST, 9449T
	1:500		1:500	1:800	1:500	1:1,000
Reference	Human (Gay et al., 2015) Mouse (Telerman et al., 2017)		Mouse (Saxena et al., 2019)	Human (Gay et al., 2015) Mouse (Fantauzzo et al., 2012)	Human (Yang et al., 2014; Purba I, Haslam et al., 2015)	Mouse (Liu et al., 2019)

*DAPI (Abcam, ab104139) for nuclear, secondary antibodies coupled to Alexa488 (Abcam, ab150077, 1:200), Alexa549 (Abcam, ab150116, 1:200), or Alexa647 (Abcam, ab150077, 1:200).*

All dorsal skin samples were embedded in paraffin, sectioned at 4 μm for the transverse and longitudinal sections. The protocol for the immunofluorescence and H&E (Sigma-Aldrich, St. Louis, MO, United States) staining was as described in Nguyen et al. (2018). Sections were imaged using the TCS SP5 confocal microscope (Leica, Wetzlar, Germany) using × 10, × 20, or × 40 objectives.

## Results

### Classification and Recognition of Hair Types in Pig

As shown in Figure 1B, four different hair types – Awl, Guard, Auchene, and Zigzag – present in the mouse could also be found in pig. The upper, middle, and bottom of back, epigastrium, and hypogastrium, collectively called the Back region, shared the same hair types. Among the four hair types, Guard (divided into Guard_1/Guard_2, discussed later) was the most abundant (32–100%) amongst all hair types, while Auchene and Zigzag only represented 4–10% and 2–8% (except 52% Zigzag hair on head) in different body parts. All four hair types were identified on the back, limb, hoof and shoulder, while buttocks, head, eyebrow, and beard contained two to three hair types; the ear and eyelash only contained Guard and Awl hair, respectively.

The quantification of HF length and HF thickness in the four hair types in pig was significantly different (Figure 1C). Comparing the two subtypes of Guard hair with the other hair types revealed that Guard_1 had the most length (41.074 ± 4.945 mm, *p* < 0.001) and thickness (37.895 ± 3.423 μm, *p* < 0.001), Guard_2 was the shortest (27.857 ± 5.201 mm, *p* < 0.001), and Zigzag had the least thickness (22.909 ± 4.300 μm, *p* < 0.001). The hair density of the head (49.333 ± 3.512/cm^2^) and back (45.667 ± 2.082/cm^2^) was significantly higher (*p* < 0.01) than that of the other body parts (Figure 1D). No significant difference in HF density was found between the limb (14.000 ± 1.000/cm^2^) with shoulder (17.333 ± 2.517/cm^2^) and hoof (23.667 ± 3.215/cm^2^) with (25.000 ± 1.732/cm^2^).

### Identification of Distinct Stages in Pig Hair Follicle Morphogenesis

Hair follicle morphogenesis development was observed at 11 different embryonic (E) and six postnatal (P) days (Supplementary Figure 2). According to the characteristic derivatives and expression of marker genes in different regions of the HF at different time points, pig HF morphogenesis development started from E37 and ended at P45. Eight key time points (E37, E41, E52, E60, E85, E107, P2, and P45) were selected to explore additional details of HF morphogenesis development and are described below.

**FIGURE 2:**
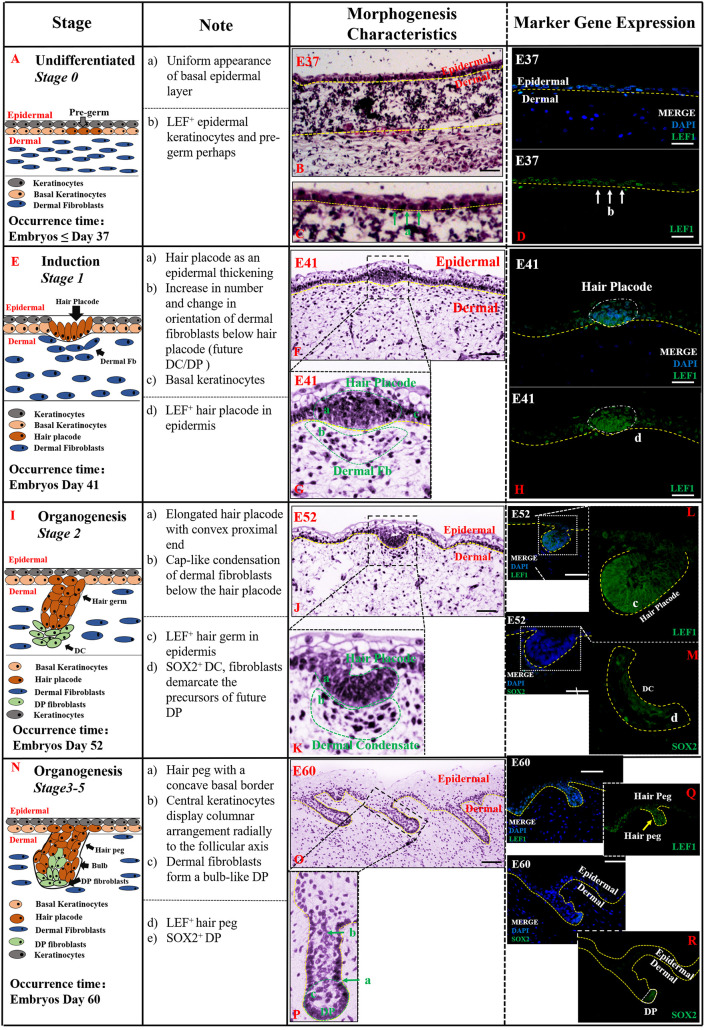
The identification stage 0 (Undifferentiated), stage 1 (Induction), and stage 2–5 (Organogenesis) in pig hair follicle morphogenesis. A schematic diagram of HF morphogenesis in stage 0 at E37 **(A)**, stage 1 between E41 and E52 **(E)**, stage 2 (E52–E60) **(I)**, and stages 3–5 (E60–E85) **(N)** between E52 and E85. The Note column summarizes simple basic parameters for HF staging by H&E staining (above dotted line), and marker gene expression (below dotted line). The characteristic of pig hair follicle morphology by H&E at E37 **(B)**, E41 **(F)**, E52 **(J)**, and E60 **(O)**. **(C,G,K,P)** Partial enlarged detail of hair follicle in parts **(B,F,J,O)**, respectively; LEF1 expression in pig hair follicle morphology at E37 **(D)**, E41 **(H)**, E52 **(L)**, and E60 **(Q)** by IF; *Sox2* expression in pig DC at E52 **(M)** and pig DP at E52 **(R)** by IF. Green and white arrows represent basal keratinocytes; yellow arrows represent hair peg. Scale bars: 50 mm.

**Undifferentiated (*Stage 0*,≤E37)** Figure 2 indicates that the first stage of HF morphogenesis development of pig occurs before E37, also termed as *pre-germ* stage. Figure 2A demonstrates a schematic diagram of the characteristic structures of the skin and HFs in this undifferentiated stage. The epidermis and dermis of skin were sharply demarcated, and basal epidermal cells were in a uniform layer. No morphological signs or molecular distinction patterns of HF were recognized in the skin (Figures 2B,C). Only *LEF1* was strongly expressed in epidermal keratinocytes in the basal and suprabasal cell layers of the epidermis (Figure 2D).

**Induction (*Stage 1*, E41–E52)** Stage 1 of HF morphogenesis is also termed as the *induction stage*. The representative characteristic structures of this stage are the formation of the hair placode (Figure 2E). Our results demonstrate that stage 1 of pig HF morphogenesis took place at around E41; the epidermal keratinocytes assembled in an upright position, and started proliferating and thickening to form the hair placode, as illustrated in Figures 2F,G. The hair placode showed strong expression of *LEF1*, whereas no expression was deleted in dermal fibroblasts (Figure 2H). Additionally, another less obvious characteristic of HF development in stage 1 was a localized increase in the number of dermal fibroblasts under the hair placode (Figure 2G). These fibroblasts began to display a slightly altered orientation, as a preparation for their subsequent aggregation.

**Organogenesis (*Stages 2–5*, E52–E85)** The organogenesis stage of HF morphogenesis is usually recognized based on the characteristic structure of hair germ and dermal condensate (DC) in stage 2 (Figure 2I), and hair peg and DP at stages 3–5 (Figure 2N). Stage 2 occurred at E52 as illustrated in Figures 2J,K. At this stage, the hair placode became more prominent and enlarged with a broad column called hair germ, which was primarily a consequence of massive epithelial cell proliferation in a well circumscribed patch of basal layer epidermal keratinocytes (Figure 2J). Few mesenchymal cells in the dermis were found condensed into a cap-like condensation and finally formed the DC (Figure 2K). *LEF1* and *SOX2* genes were strongly expressed in the enlarged epithelial hair germ and DC, respectively (Figures 2L,M).

Stages 3–5 were found to be the continuation of stage 2, and the HF morphogenesis in these three stages happened at around E60–E85. Based on the characteristic structures of HF, at this stage, hair germ and DC had differentiated into hair peg and dermal papilla (DP) (Figure 2N). The hair peg is generally identified by large, tightly packed vertically oriented keratinocytes which directly invaginate into the dermis (Figure 2O). The DC remains on the leading edge of the down-growing hair peg and, upon engulfment by the HF matrix, transitions into a bulb-like DP (Figure 2P). Like the previous stages, *LEF1* and *SOX2* were more strongly expressed on hair peg and DP, respectively (Figures 2Q,R).

**FIGURE 3:**
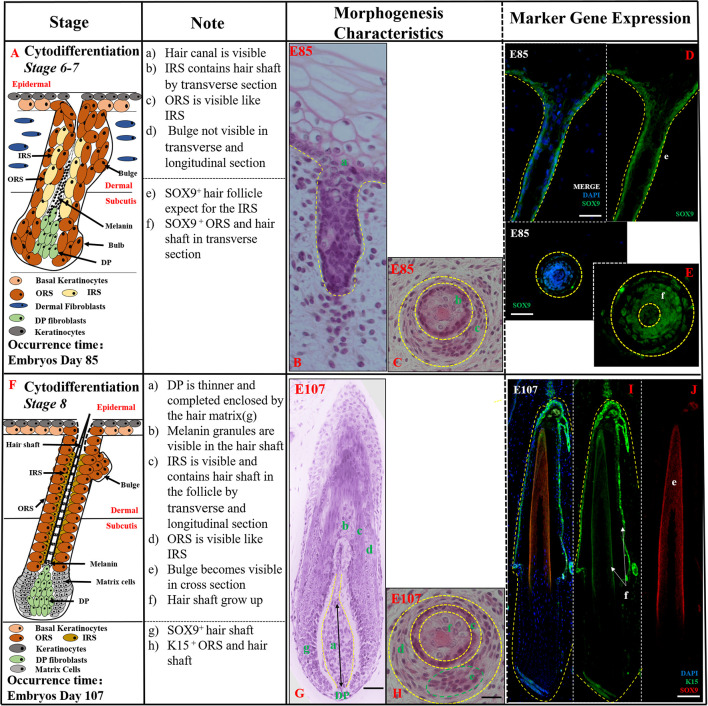
The identification of stages 6–8 (Cytodifferentiation) of pig hair follicle morphogenesis. **(A,F)** A schematic diagram of stages 6–7 and stage 8 in HF morphogenesis after E85. The Note column summarizes simple basic parameters for HF stage by H&E staining (above dotted line) and marker gene expression (below dotted line). **(B)** The characteristics of pig hair follicle morphology at E85 by H&E. **(C)** Partial enlarged detail of hair follicle in part **(B)**; *SOX9* expression in ORS and hair shaft at E85 in longitudinal **(D)** and transverse **(E)** section by IF. **(G)** The characteristics of pig hair follicle morphology at E107 by H&E. **(H)** Partial enlarged detail of hair follicle in part **(G)**. **(I)** *K15* expression in pig ORS and hair shaft at E107 by IF. **(J)** *Sox9* expression in pig hair shaft at E107 by IF. Scale bars: 50 mm.

**Cytodifferentiation (*Stages 6–8*,>E85)** In this period, the mature HF was almost developed while inner root sheath (IRS), outer root sheath (ORS), and hair shaft were visible (Figures 3A,F). In stages 6–7 (E85-E107), the hair peg resembled a mature HF more closely. The DP was completely engulfed by the surrounding matrix cells at the base of the hair peg, the IRS elongated up through the developing follicle, and the hair canal development had started (Figure 3B). The hair shaft, IRS, and ORS were visible in transverse section (Figure 3C). Moreover, the mature ORS and hair shaft were recognized by SOX9-stained hair keratins, while the IRS, derived from DP, showed almost negative expression (Figures 3D,E).

In stage 8 (>E107), the HF had already matured, and the key characteristic structures were hair canal visibility and the hair shaft formation. The tip of the hair shaft exited the IRS, entered the hair canal, and emerged through the epidermis (Figure 3G). The hair shaft, IRS, ORS, and CTS were all clearly visible in transverse sections (Figure 3H). The expression of *K15* was detected in the ORS (Figure 3I) and hair shaft whose precursor cells coming from epidermis basal keratinocytes had strong expression of *SOX9* (Figure 3J). Morphologically, the HF was mature and resembled an early-stage anagen HF.

### Prenatal Hair Follicle Morphogenesis Development Maintained Until Postnatal P45

**FIGURE 4:**
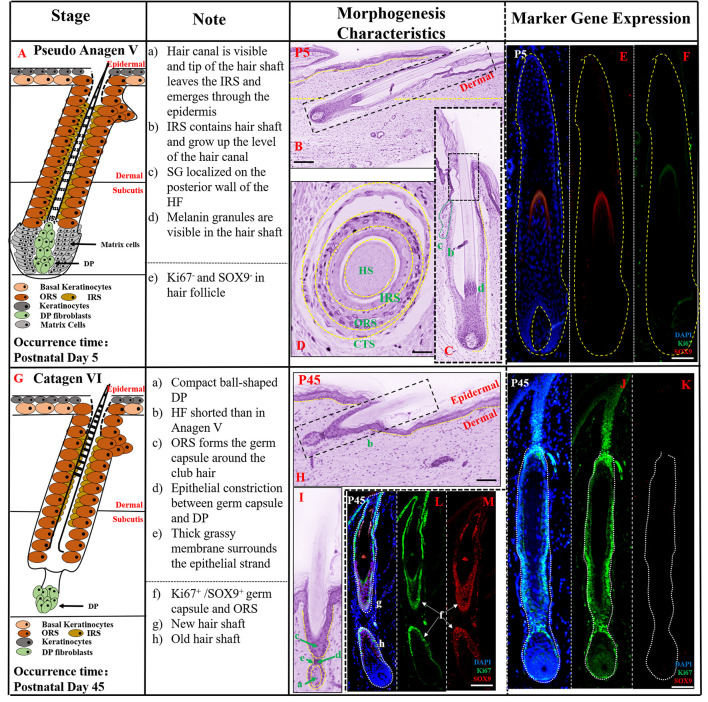
The prenatal hair follicle morphogenesis development maintained until postnatal P45. **(A,G)** A schematic diagram of HF morphogenesis development in the postnatal stage (P5–P45). The Note column summarizes simple basic parameters for HF staging by H&E staining (above dotted line) and marker gene expression (below dotted line). **(B)** The characteristics of pig hair follicle morphology at P5 by H&E. **(C)** Partial enlarged detail of hair follicle in part **(B)**. The characteristics of pig hair follicle morphology in transverse section at P5 **(D)** and P45 **(H)** by HE; **(E)** *SOX9* expression in hair shaft at P5 by IF; **(F)** *Ki67* expression in proliferation keratinocyte cells at P5 by IF; **(I)** Partial enlarged detail of hair follicle in part **(H)**. **(J,L)** *Ki67* expression in pig proliferation keratinocytes cells at P45 by IF. **(K,M)** *Sox9* expression in pig hair follicle at P45 by IF. Scale bars: 50 mm.

According to our observations of HFs at several periods after E107 (Supplementary Figure 1), HF morphogenesis development was maintained until P45, and then the first stage of HF cycle development commenced – the catagen stage. The period of E107-P45 resembled a pseudo-anagen stage as the hair follicle showed continued development and the characteristic morphology was similar to anagen V in P5 (Figure 4A). In this stage, the tip of the hair shaft grew through the hair canal and epidermis as seen in longitudinal section (Figure 4B) while the connective tissue sheath (CTS), ORS, IRS, and hair shaft formed a cylinder from outside to inside in transverse section (Figure 4D). In addition, the sebaceous gland (SG) had located on the side of the HF (Figure 4C). As the indicator of proliferation ability of keratinocyte cells, the expression of *SOX9* and *Ki67* at P5 was reduced, compared with their expression at E107 (Figures 4E,F), indicating the slowing down of HF morphogenesis development and gradual entry into the final stages of development.

At P45, the key characteristic structure of catagen period is the beginning of atrophy of the DP to a ball-shaped structure (Figure 4G). As shown in Figures 4H,I, HF length at P45 was significantly shorter than in pseudo anagen V, closer to the epidermis and ORS forming the germ capsule around the club hair. The HF displayed another catagen characteristic, the presence of an epithelial strand between the secondary germ capsule and the DP. Furthermore, no *SOX9* expression was detected on the hair shaft (Figure 4K) and strong expression of *Ki67* was demonstrated in the HF (Figure 4J), indicating that the HF was inactive and had entered the catagen stage. Interestingly, the characteristic of pseudo-anagen IIIb stage was also detected at this stage; the newly formed hair shaft was fully enclosed by the IRS, while the older hair shaft had moved up. Furthermore, strong expression of *SOX9* (Figure 4M) and *Ki67* (Figure 4L) in ORS and contraction of matrix cells were identified, suggesting the commencement of the first pseudo-anagen period in HF morphogenesis.

## Discussion

**FIGURE 5:**
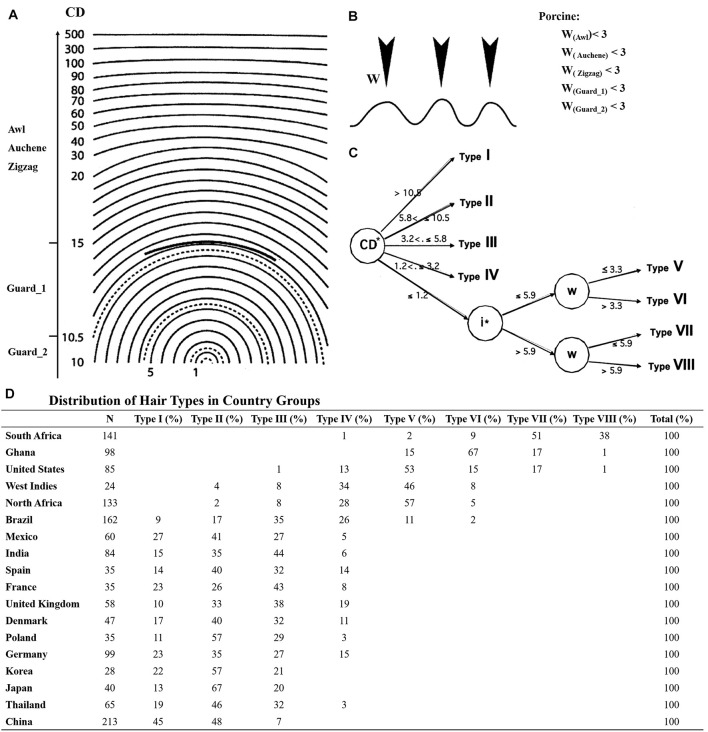
The hair types between pig and human scalp hair. **(A)** CD meter used in the determination of five pig hair type curve diameter. **(B)** Number of waves (W) in five pig hair types. **(C)** Segmentation tree of eight human scalp hair; CD meter template derived from Bailey and Schliebe (1985); the graphics of the waves in **(B)** derived from De la Mettrie et al. (2007). **(D)** Distribution of hair types in country groups.

Although the mouse is the most commonly used animal model for research on human HF development, the classification of hair types in human has been implemented in different ways, mainly depending on the body parts (e.g., scalp, beard, chest, pubic, and axillary hair) with scalp hair being the most studied hair type (Duverger and Morasso, 2009; Buffoli et al., 2014). Nonetheless, similarities could still be explored between pig and human scalp hair. De la Mettrie et al. (2007) collected hair samples from 18 countries and classified eight main types of scalp hair based on curve diameter (CD), waves (w), and twists (i). In this study, all four different hair types were identified in pig referred to mouse. However, based on the hair shaft length and diameter, Guard hair in pig was further divided into two different subtypes of Guard_1 and Guard_2. Guard_2 had a larger angle bending and shorter length. In comparison with the CD and w of human scalp hair (Figure 5A), the CDs of pig Awl, Auchene, and Zigzag hair types were greater than 20 and the CD of Guard_1 was 10.5–15. Besides, they were similar with Type I human scalp hair reported by De la Mettrie et al. (2007), while the CD of Guard_2 was 5.8–10.5 like Type II in human scalp hair (Figure 5A). According to waves (w), human scalp hair was further divided into four other types (Figure 5C), while the w of all pig hair types was less than 3 μm (Figure 5B).

Taken together, Guard_2 showed higher similarity to type II human scalp hair while other pig hair types (Guard_1, Awl, Auchene, and Zigzag) were similar to type I of human scalp hair. More interestingly, the type I and II human scalp hair makes up a large proportion of eight different human scalp hair types, particularly for Asians and Europeans, and type II was in higher proportion than type I in all samples (Figure 5D). However, types III–VIII of human scalp hair were not detected in our study mainly because only Yorkshire pig was used. Other pig breeds with curly/wool hair such as the Mangalica pig (Schachler et al., 2020), Turopolje pig (Čandek-PotokarRosa and Nieto, 2019), Canastrao pig (Rhoad, 1934), or Cuino pig (Lemus-Flores et al., 2005) could be used to identify more hair types. Thus, considering the similarity of the pig hair types to human scalp hair, pig proves to be an ideal model for human HF research.

In addition to hair types, pig and human hair also demonstrated similarity in hair thickness and hair density (Table 2). The hair thickness of pig (18–49 μm) is closer to human (16–42 μm) (Otberg et al., 2004) and the hair density of back (24–28/cm^2^) and limb (13–27/cm^2^) hair of pig in this study was very close to that of human back (29/cm^2^) and limb (14–32/cm^2^) (Otberg et al., 2004). Although the hair density of human scalp hair (292/cm^2^) was much higher than pig head (46–53/cm^2^), the trend of a higher head HF density than other body parts was shared by pig and human (Table 2 and Figure 1D). Unlike mice, which exhibit pelage with synchronous growth patterns, the asynchronous hair growth in different human body parts (Buffoli et al., 2014) leads to more growth of the HFs on the scalp and face than on the arms and legs (Seago and Ebling, 1985; Pagnoni et al., 1994). Thus, based on our results which showed similarity of hair type and growth pattern between pig and human, it could be speculated that the pig hair growth is also asynchronous (Koch et al., 2020).

**TABLE 2:** Hair characteristic parameters between human and pig.

Species	Hair type	Hair thickness (μm)*	Hair density (/cm^2^) *			References
			Head	Back	Limb^1^	
Human	*Type I –Type VIII*	16–42	292	29	14–32	Otberg et al., 2004; De la Mettrie et al., 2007; Buffoli et al., 2014
Pig	*Awl, Guard_1, Guard_2, Auchene, Zigzag*	18–49	46–53	24–28	13–27	**Our study**

*Hair thickness and hair density in human was based on scalp hair; ^1^human limb contains upper arm, forearm, thigh and calf.

**FIGURE 6:**
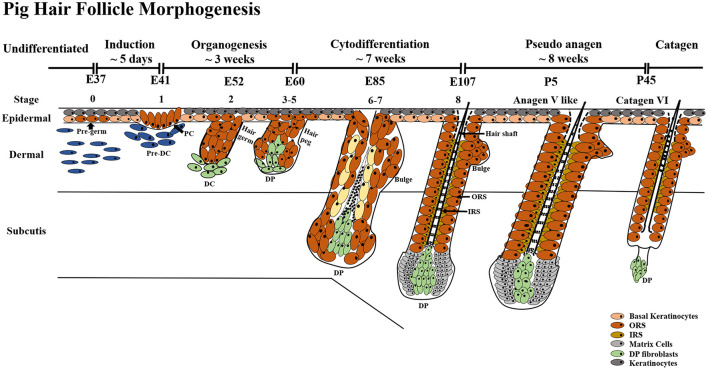
HF morphogenesis development during pig embryogenesis. HF morphogenesis development in the prenatal stage is critically important, which determines HF cycle development in the postnatal stage. Pig HF morphogenesis development has been summarized into three main periods from E41 to P45 – induction (E37–E41), organogenesis (E41–E85), and cytodifferentiation (>E85). Stages 1–8 of embryonic hair development are depicted, demonstrating the continuous transition between hair follicle development and the first postnatal hair cycle. PC, hair placode; DC, dermal condensate; DP, dermal papilla; IRS, inner root sheath; ORS, outer root sheath.

Based on 11 different embryonic days and 6 postnatal days, our results indicated that pig HF morphogenesis development started from E37 and ended at P45. According to eight key time points from E37-P45, we further summarized three periods of the HF morphogenesis development during embryogenesis, induction (E37–E41), organogenesis (E41–E85) and cytodifferentiation (>E85) (Figure 6).

Generally, prenatal HF morphogenesis development has been divided into three main periods. The morphological characteristics of hair placode, DC, DP, etc., at different periods are nearly the same in human (Muller et al., 1991), mice (Schneider et al., 2009), sheep (Zhang et al., 2019), rabbit (Ding et al., 2020) and, based on the current study, even in pig. However, the hair type and commence time points of each HF morphogenesis development stage has been shown to vary largely, e.g., secondary HF in sheep and rabbit could make the HF morphogenesis complicated, thus making them not ideal model systems for human HF research.

As summarized in Table 3, fetal HF induction occurs in about the 12th week, organogenesis in the 14th week, cytodifferentiation in the 16th week, and mature hair in the 18th week of gestation (Muller et al., 1991). Comparing the average gestation period of human (∼40 weeks), pig (∼16 weeks), and mouse (∼21 days), it can be concluded that prenatal HF morphogenesis development goes through in the early/mid gestational stage in human (∼12 weeks) and pig (∼6 weeks), while in mouse, mid/late gestational stage occurs (∼14 days). Moreover, hair shafts emerge through the epidermis before birth in both human and pig, while the mouse hair shaft is not visible until 1 week after birth (Harland, 2018). These differences are reflected consistently in human and mouse HF studies. According to the Duverger and Morasso (2009), the first HF cycle begins from the first catagen stage after birth. To explore the entire HF morphogenesis in pig, HF development in postnatal stages was also investigated in the current study. Our results showed that most HFs enter catagen at P45 while some are kept in pseudo anagen V. This phenomenon might be a result of the asynchronous HF development of different hair types. Similar asynchronous HF development is also found in human; the normal ratio between active anagen hairs and resting telogen hairs is 85/15 in human scalp hair (Robertson, 1999) because of the asynchronous hair growth and development periods in different body regions. However, the different hair types in mouse coat were synchronous in growth (Koch et al., 2020). In the current study, we have only provided a rough time point for each of the HF morphogenesis development stages; further research is needed for a better understanding of the different time points in pig HF morphogenesis.

**TABLE 3:** Classification of the hair follicle morphogenesis periods in different species.

Species	Induction	Organogenesis	Cytodifferentiation	Mature hair	Pregnancy period	Hair visible in birth	HF starting stage*	References
Human	12th weeks∼	14th weeks∼	16th weeks∼	18th weeks∼	∼40 weeks	Yes	Early/Mid	Muller et al., 1991; Chang et al., 2005
Pig	6th weeks∼	7th weeks∼	12th weeks∼	15th weeks∼	∼16 weeks	Yes	Early/Mid	**Our study**
Mouse	E13.5–14.5	E15.5–17.5	E18.5	P3	∼21 days	No	Mid/Late	Duverger and Morasso, 2009

*HF begins morphogenesis development in the embryo stages. Early, early gestational stage; Mid, middle gestational stage; Late, late gestational stage.

Over the past several decades, the pig has become an increasingly popular biomedical model especially in skin research. Both human and pig show abundant subdermal adipose tissue (Morris and Hopewell, 1990), similar regeneration time of epidermal cells (30 day for pig vs. 27–28 day for human) (Weinstein, 1965), and thickness of epidermis (30–140 nm for pig vs. 50–120 nm for human) (Meyer et al., 1978). The marker genes (Table 1) also showed consistent expression patterns in pig in comparison to human and mouse. Our previous study also shows that other classical pathway marker genes like *Wnt5a*, *CTNNB1*, *DKK4*, and *BMP4* reported in mouse (Saxena et al., 2019) and human HF (Koch et al., 2020) were also being expressed in hair placode formation during the induction and organogenesis stages in pig (Jiang et al., 2019). Thus, collectively, our study demonstrates the shared similarities in hair types and HF morphogenesis development pattern between pig and human, further bolstering our hypothesis about the pig being an ideal model for HF research.

In summary, in this study, we explored the various hair types and classification of distinct stages of HF morphogenesis in pig by their morphology and marker gene expression. Our findings indicated that pig and human show a higher similarity in hair types and HF morphogenesis patterns. Thus, apart from the pig being a better model to decipher human HF morphogenesis, it also offers the opportunity to researchers of being a new model of hair research. Although in this study we pay more concern to the pig HF morphogenesis development in embryogenesis, it is also plausible to use pig as an ideal animal model to investigate human hair cycle after birth due to the similarity of pig hair types, gene expression patterns, and asynchronous growth patterns similar to human in postnatal stages, although further studies are necessary to confirm these aspects.

## Data Availability Statement

The original contributions presented in the study are included in the article/Supplementary Material, further inquiries can be directed to the corresponding author.

## Ethics Statement

The animal study was reviewed and approved by the Institutional Animal Care and Use Committee of China Agricultural University.

## Author Contributions

XD and YJ: conceptualization. XD: methodology, writing, review, editing, and funding acquisition. YJ and QZ: formal analysis. SL and YW: investigation. QZ: visualization. BL and TL: resources. YJ: writing – original draft preparation. All authors contributed to the article and approved the submitted version.

## Conflict of Interest

The authors declare that the research was conducted in the absence of any commercial or financial relationships that could be construed as a potential conflict of interest.

## Publisher’s Note

All claims expressed in this article are solely those of the authors and do not necessarily represent those of their affiliated organizations, or those of the publisher, the editors and the reviewers. Any product that may be evaluated in this article, or claim that may be made by its manufacturer, is not guaranteed or endorsed by the publisher.

## Supplementary Material

Supplementary Figure 1 Specificity detection of five antibodies in pig hair follicle. FBS: negative control, fetal bovine serum substitute antibody.

Supplementary Figure 2 H&E staining of Hair follicle morphogenesis at 11 different prenatal and six postnatal times (E37–P45). The red arrow represents the hair placode and hair germ. The green arrow represents the hair peg. The Blue arrow represents the (1) outer root sheath, ORS; (2) inner root sheath, IRS; (3) hair shaft; (4) dermal papilla, DP. The red dotted line represents the critical period of hair follicle morphogenesis development; Scale bars, 200 μm.

## Abbreviations

HF: hair follicle
DC: dermal condensate
DP: dermal papilla
IRS: inner root sheath
ORS: outer root sheath
SG: sebaceous gland
CTS: connective tissue sheath
AP: alkaline phosphatase activity
IL-1-RI: interleukin-1 receptor type 1
TGF- β-RII: transforming growth factor β receptor type II
SOX2: SRY-box transcription factor 2
LEF1: lymphoid enhancer binding factor 1
SOX9: SRY-box transcription factor 9
KRT15: keratin 15
Wnt5a: Wnt family member 5A
CTNNB1: catenin beta 1
DKK4: Dickkopf WNT signaling pathway inhibitor 4
BMP4: bone morphogenetic protein 4.
