# Supplementary figures and images for: Atlas of Prenatal Hair Follicle Morphogenesis Using the Pig as a Model System
Source: Front Cell Dev Biol. 2021 Oct 7;9:721979. doi: 10.3389/fcell.2021.721979 (PMC8529045; doi:10.3389/fcell.2021.721979)

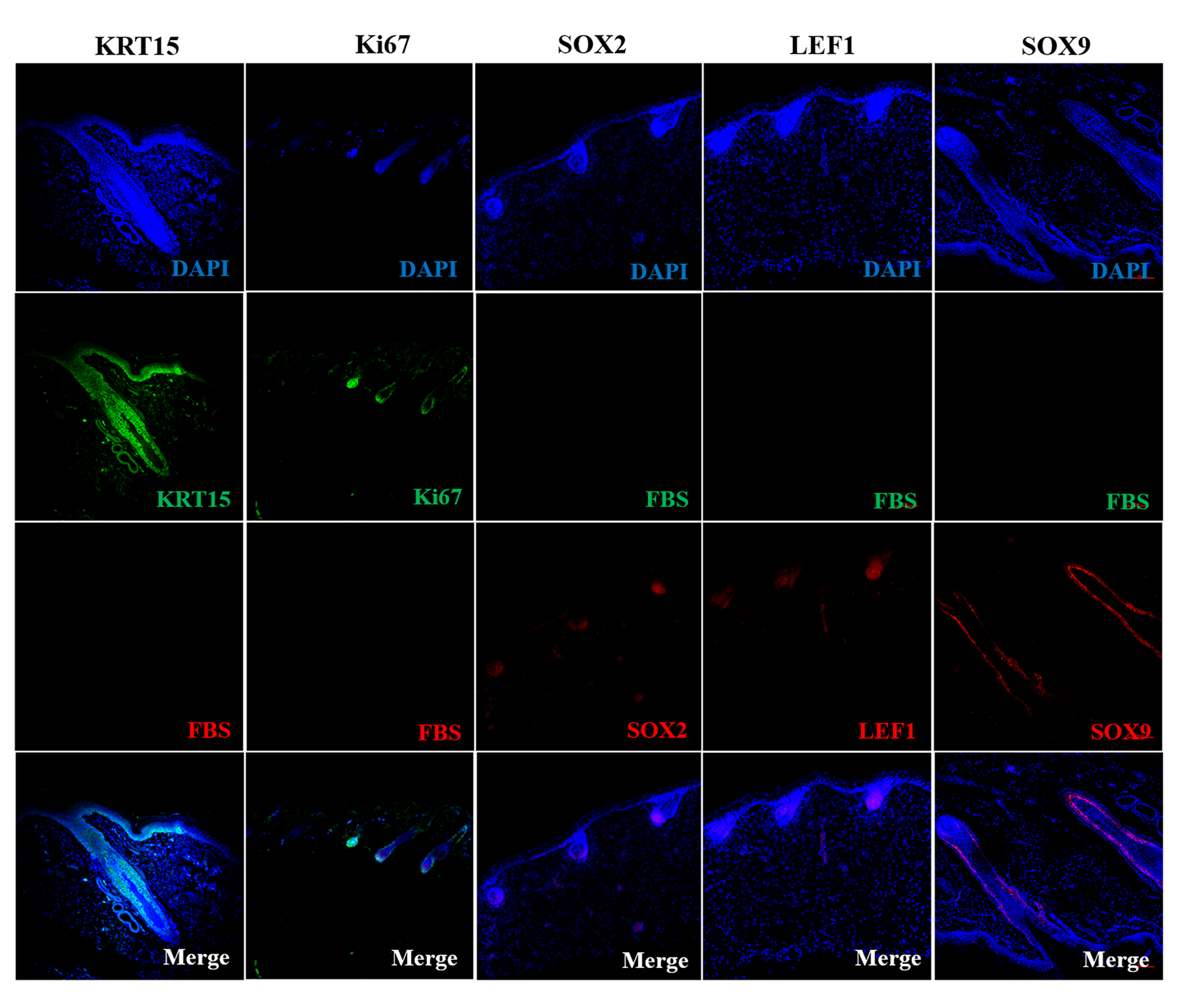

Supplement: Supplementary Figure 1 — Specificity detection of five antibodies in pig hair follicle. FBS: negative control, fetal bovine serum substitute antibody. [file Image_1.TIF]

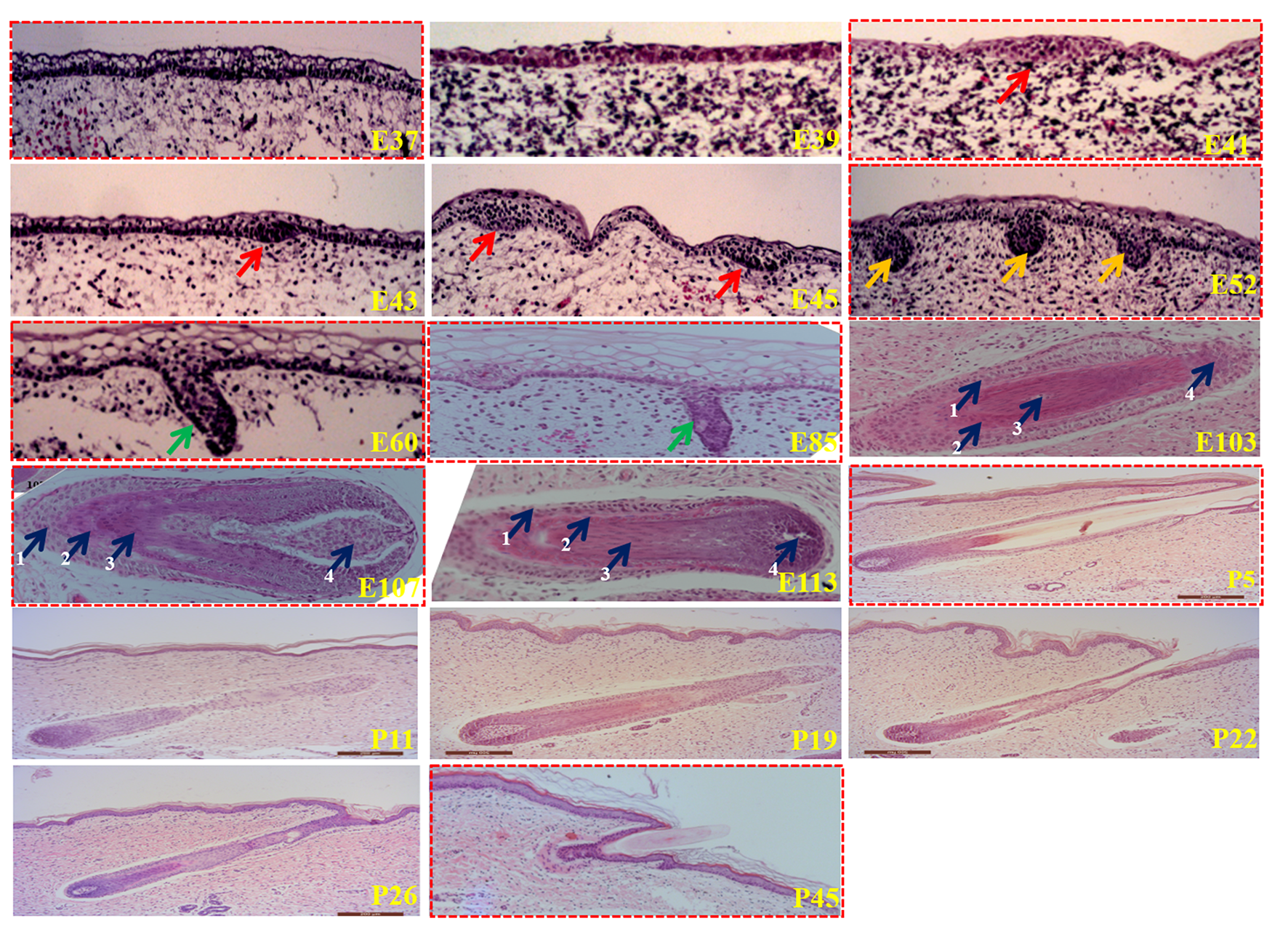

Supplement: Supplementary Figure 2 — H&E staining of Hair follicle morphogenesis at 11 different prenatal and six postnatal times (E37–P45). The red arrow represents the hair placode and hair germ. The green arrow represents the hair peg. The Blue arrow represents the (1) outer root sheath, ORS; (2) inner root sheath, IRS; (3) hair shaft; (4) dermal papilla, DP. The red dotted line represents the critical period of hair follicle morphogenesis development; Scale bars, 200 μm. [file Image_2.TIF]
